# Key Characteristics of Nitrous Oxide-Induced Neurological Disorders and Differences Between Populations

**DOI:** 10.3389/fneur.2021.627183

**Published:** 2021-04-27

**Authors:** Juanjuan Zhang, Dandan Xie, Yanfeng Zou, Xuen Yu, Yang Ji, Chengyou Wang, Xinyi Lv, Nong Zhou, Xiao Jiang, Kai Wang, Yanghua Tian

**Affiliations:** ^1^Department of Neurology, The First Affiliated Hospital of Anhui Medical University, Hefei, China; ^2^Department of Neurology, The Affiliated Anqing Hospital of Anhui Medical University, Anqing, China; ^3^Department of Epidemiology and Biostatistics, School of Public Health, Anhui Medical University, Hefei, China; ^4^Department of Neurology, The Institute of Neurology of Anhui University of Chinese Medicine, Hefei, China; ^5^Anhui Medical University, Hefei, China; ^6^Department of Neurology, The Affiliated Tongling Hospital of Anhui Medical University, Tongling, China; ^7^Department of Neurology, The First Affiliated Hospital of the University of Science and Technology of China, Hefei, China; ^8^The School of Mental Health and Psychological Sciences, Anhui Medical University, Hefei, China; ^9^Anhui Province Key Laboratory of Cognition and Neuropsychiatric Disorders, Hefei, China; ^10^Collaborative Innovation Center for Neuropsychiatric Disorders and Mental Health, Hefei, China; ^11^Institute of Artificial Intelligence, Hefei Comprehensive National Science Center, Hefei, China

**Keywords:** nitrous oxide, vitamin B12, homocysteine, spinal cord disease, peripheral nervous system diseases

## Abstract

**Background:** Nitrous oxide (N_2_O), commonly known as laughing gas, is inhaled recreationally because it produces the feelings of euphoria and freedom from pain. The risk of neurological dysfunction secondary to N_2_O abuse and its clinical diagnosis are, however, not yet sufficiently recognized, especially in China. Here, we have summarized the key clinical characteristics of N_2_O-induced neurological disorders.

**Materials and Methods:** We recruited 20 patients with N_2_O-induced neurological disorders and analyzed their clinical features, laboratory data, magnetic resonance imaging and electromyography. We also carried out a literature review and compared 99 previously reported patients with our case series to confirm our results. Subgroup analysis was performed to explore the difference in demographical and clinical characteristics of N_2_O abuse between Asian and non-Asian patients.

**Results:** The most common initial symptoms of N_2_O-induced neurological disorders were weakness and/or paresthesia. Most patients presented with myelopathy and/or peripheral neuropathy. The most commonly involved segment of the spinal cord was the cervical spinal cord, extending over 4–6 vertebral levels, but more than half of the patients with myelopathy had no sensory change at the corresponding spinal level. Homocysteine was found to be the most sensitive and practical indicator for diagnosis. Subgroup analysis showed that the Asian patients (median: 22.0 years old, Q1–Q3:19.0–26.0 years old) with N_2_O abuse were younger than non-Asian patients [26.0 (22.3–31.0) years old, *P* = 2.8 × 10^−4^]. The incidence of myelopathy combined with peripheral neuropathy was significantly higher in Asian patients than in non-Asian patients, who had myelopathy or peripheral neuropathy (*P* = 2 × 10^−5^).

**Conclusions:** Key clinical characteristics of N_2_O abuse are longitudinally extensive cervical myelopathy and peripheral neuropathy. Recognition of these traits in young people in the age group of 20–30 years will provide important guidance for accurate diagnosis of neurological disease associated with N_2_O abuse. The clinical manifestations differ in Asian patients and non-Asian patients.

## Introduction

Nitrous oxide (N_2_O), an odorless and colorless gas, which is also known as laughing gas, is widely used in anesthesia and as a propellant in the food industry (1, 2). Unfortunately, because of its legitimation, convenience and low cost, N_2_O is recreationally inhaled by people for the feelings of euphoria and freedom from pain (3, 4). N_2_O abuse has now become a common societal and medical problem in many countries, including the USA, UK and China (1, 3, 5) According to the Global Drug Survey of 2016, N_2_O is the seventh most commonly used recreational drug (6). Since the first Chinese case was reported in 2016, there has been a rapid annual increase in published papers related to recreational abuse of N_2_O in China (3). The risks associated with N_2_O abuse and its clinical diagnosis are, however, not yet sufficiently recognized, especially in China.

Alterations in vitamin B12 (methylcobalamin and adenosylcobalamin) metabolism were speculated to underlie the neurotoxic effects of N_2_O (4, 7–9). Methylcobalamin converts homocysteine to methionine as part of the methylation cycle. The process might be disturbed by N_2_O via irreversible oxidation of the cobalt atom of vitamin B12, which leads to low levels of vitamin B12, hyperhomocysteinemia, demyelination, axonal degeneration, and megaloblastic anemia (10). Adenosylcobalamin is a cofactor for L-methylmalonyl coenzyme A (MMCoA) mutase, which catalyzes the conversion of methylmalonyl-CoA to succinyl-CoA in mitochondria. Inactivation of adenosylcobalamin results in reduced levels of succinyl-CoA, and high levels of methylmalonic acid (MMA) which also leads to demyelination (9).

Individuals who abuse N_2_O may present with numbness, weakness, ataxia, decreased deep sensation, urinary retention, sleep disorders, hallucinations or even death (4, 7, 11–13). However, these neurological symptoms secondary to N_2_O abuse are non-specific and often mimic other diseases (14). If the patient conceals, or the neurologist ignores, recreational N_2_O abuse, the patient may be wrongly diagnosed with autoimmune diseases, such as Guillain-Barre syndrome (GBS) and receive unnecessary treatments (i.e., intravenous immunoglobulin, IVIG). It is thus important that clinicians recognize the clinical features of neurological disease associated with N_2_O abuse.

Here, we recruited 20 patients diagnosed with N_2_O-induced neurological disorders and summarized their clinical features, laboratory data, magnetic resonance imaging (MRI) and electromyography (EMG). We also carried out a literature review and compared 99 previously reported patients with our case series to confirm our results. Homocysteine metabolism is involved in vitamin B12 metabolism and might differ among various populations. To explore the demographical and clinical characteristics of N_2_O abuse between Asian and non-Asian patients, we pooled the data for our 20 patients and the previously reported 99 patients to conduct subgroup analysis (15).

## Materials and Methods

This retrospective hospital-based case series study was conducted in the First Affiliated Hospital of Anhui Medical University, the Affiliated Anqing Hospital of Anhui Medical University, the Institute of Neurology of Anhui University of Chinese Medicine, the First Affiliated Hospital of the University of Science and Technology of China and the Affiliated Tongling Hospital of Anhui Medical University. We searched the hospital medical records from October 2018 to May 2020 to identify patients (≥14 years old) with a history of N_2_O abuse and symptoms suggestive of neurological disease using an institutional search tool. Patients with myelopathy and/or peripheral neuropathy caused by infection, autoimmunity, trauma, vascular disease, other toxic/metabolic causes, tumor, heredity and systemic disease were excluded. One patient had a pre-existing diagnosis of N_2_O-induced subacute degeneration of the spinal cord (SCD) and had re-inhaled N_2_O before this admission (case 20). None of the patients were vegetarian or had undergone gastrectomy and so were unlikely to have vitamin B12 deficiency. It was not possible to obtain consent from all patients since current contact details were unavailable and all cases were, therefore, anonymized. The Clinical Research Ethics Committee of Anhui Medical University approved this study protocol, which was in accordance with the Declaration of Helsinki. Patients gave written informed consent.

Details of clinical symptoms, examinations and treatment and prognosis of these patients were collected. Because standard techniques and routine treatment varied between the different hospitals, some data were missing.

To further demonstrate clinical characteristics of N_2_O abuse, we performed a systematic literature review through searches of MEDLINE and EMBASE, with the key words (nitrous oxide or laughing gas or N_2_O) AND (neurological disease or myelopathy or peripheral neuropathy or Guillain-Barre syndrome or GBS or subacute degeneration of the spinal cord or SCD or degeneration or spinal cord or neurotoxicity), with the studies limited to human and case reports limited to the period January 1998 to July 2020. Cases before 1998 were not included due to undocumented spinal cord MRI. Studies without detailed documentation of patients' clinical features were excluded, even if they had a large sample size. Exclusion criteria were: non-English language publication, patients younger than 14 years old, symptoms caused by infection, autoimmunity, trauma, vascular disease, other toxic/metabolic causes (such as N_2_O anesthesia or vegetarian diet), tumor, heredity and systemic disease. The search yielded previously reported 99 patients were recruited. We extracted the following information: gender, age, clinical manifestations, examinations, treatment and prognosis. Two investigators (Zhang and Ji) independently reviewed the retrieved articles to choose potentially relevant articles and extracted data.

We pooled the data from our own 20 patients and the 99 previously reported patients then divided them into two groups: Asian patients from Asian countries and non-Asian patients from European, North American, and Oceanian countries. We created two more subgroups from pooling the data of our own 20 patients and 23 patients of published cases from China. These two groups were patients from mainland China and patients from Taiwan. After reassigning the cases, we performed analyses to explore the differences in demographical and clinical characteristics of N_2_O abuse between each grouping pair (Asian vs. non-Asian and mainland China vs. Taiwan).

All statistical analyses were conducted using IBM SPSS (version 25.0). Normally distributed continuous variables were expressed as the mean ± standard deviation (SD) and compared with the independent sample *t*-test. Continuous variables that were not normally distributed were expressed as the median (Q1–Q3) and compared with the Mann-Whitney U test. The chi-squared test was used for comparisons of categorical variables. A probability level of <5% (two-sided) was regarded as significant.

## Results

### Demographics

Clinical features of 20 patients are summarized in Supplementary Table 1 and Table 1. All patients were young (median: 22.5 years old, Q1–Q3: 20.3–27.8 years old). Eleven were female and nine were male. The duration of N_2_O abuse was between 3 days and 2 years.

**Table 1:** Clinical features.

		**Current cohort**		**Reported patients**	
		***N***	**%**	***N***	**%**
Gender	Male/female	11/9	55.0/45.0	55/44	55.6/44.4
Age (years)		22.5 (20.3–27.8)		24.0 (21.0–29.0)	
Previous history	Mental disease	0	0	15	15.2
	Substance abuse (except N_2_O)	0	0	16	16.2
Initial symptoms	Paresthesia or numbness	17	85.0	66	66.7
	Gait	4	20.0	30	30.3
	Weakness	14	70.0	29	29.3
	Pain	0	0	4	4.0
	Bowel or bladder dysfunction	0	0	4	4.0
	Ataxia	0	0	14	14.1
	Impaired cognition	0	0	7	7.1
	Double vision	0	0	1	1.0
	Involuntary movement	0	0	1	1.0
Clinical manifestation	Paresthesia or numbness	19	95.0	86	87
	Weakness (upper/lower/four limbs)	0/6/10	0/30.0/50.0	4/31/26	4.0/31.0/26.3
	Bowel or bladder dysfunction	1	5.0	19	19.2
	Pain	2	10.0	9	9.1
	Thrombosis	1	5.0	6	6.1
	Psychiatric symptoms	2	10.0	9	9.1
	Impaired cognition	1	5.0	10	10.1
	Involuntary movement	0	0.0	7	7.1
	Decreased deep sensory	8	40.0	79	79.8
	Ataxia	11	55.0	66	66.7
	Skin change(pigmentation and erythema)	0	0.0	7	7.1
	Lhermitte's sign	0	0.0	8	8.1
Laboratory exams	Vitamin B12:decreased/normal	5/8	38.5/61.5	50/43	53.8/46.2
	Homocysteine:elevated/normal	14/2	87.5/12.5	55/4	93.2/6.8
	Hb:low/normal	3/17	15.0/85.0	20/44	31.3/68.7
	Mean corpuscular volume:elevated/normal	5/15	25.0/75.0	26/43	37.7/62.3
	Methylmalonic acid:elevated/normal	0	0	32/2	94.1/5.9
Spinal cord involvement	Cervical segment	12	80.0	52	52.5
	Cervical and thoracic segment	3	20.0	20	20.2
	Thoracic segment	0	0.0	1	1.0
	Not noted	0	0.0	3	3.0
	Without spinal sensory level	13	86.7	50	65.8
Electromyography	Axonal damage and demyelination/axonal/demyelination	10/2/4	62.5/12.5/25.0	14/13/12	31.1/28.9/26.7
	Unknown damage type	0	0	6	13.3
Treatment	Vitamin B12/steroid/IVIG/Methonine	20/6/2/0	100/30.0/10.0/0	95/8/5/4	96.0/8.1/5.1/4.0
Prognosis	Improvement/worse/remained/death	16/0/1/0*	80.0/0/5.0/0	75/1/4/1^Δ^	75.8/1.0/4.0/1.0

IVIG, intravenous immunoglobulin;/:data which were not collected; ^*^three patients were lost; ^Δ^seven patients were lost,11 patients' prognosis were not mentioned. Age was expressed as median (Q1–Q3). Patients were treated with IVIG because most of them did not admit to N_2_O abuse at the beginning of treatment. Patients were treated with steroids, either to alleviate edema of the spinal cord or because they did not admit to N_2_O abuse.

### Clinical Manifestations

All 20 patients initially presented with weakness or numbness. Eleven patients presented with both weakness and numbness. Six patients presented with only numbness. Three patients presented with only weakness, including one patient who did not complain of paresthesia but presented with abnormal deep sensation on physical examination and one patient who complained of paroxysmal numbness of distal parts of all four limbs but presented with normal sensation on physical examination. In half of the patients, weakness was present in all four limbs. In 30% of patients, weakness was present only in the lower limbs. In other patients, the whole body (1/20) or only one leg (1/20) was affected. Besides weakness and paresthesia, eleven patients presented with ataxia and eight of them with disturbances in proprioception over the course of the disease. Unsteady gait and pain were complained of in four and two patients, respectively. Psychiatric symptoms, sleep disturbance, bowel and bladder dysfunction, arterial thrombosis and memory impairment were also observed (cases 6, 12, 15, 18, 20). The cognition of patients was evaluated by mini-mental state examination, Montreal cognitive assessment, Stroop test, verbal fluency test, digit span test and Chinese auditory vocabulary learning test.

### Laboratory Data

Laboratory tests and ancillary examinations of 20 patients are summarized in Supplementary Table 1 and Table 1. The average hemoglobin (Hb) level was 133 ± 20 g/L. Three female patients showed anemia (normal: female >110 g/L; male >120 g/L). Mean corpuscular volume (MCV) was 96 ± 9 fl. Elevated MCV was found in five patients (normal <100 fl). Only five patients had deceased levels of vitamin B12 (normal: 197–771 pg/mL). High homocysteine levels were found in 14 patients (normal: 5–13.9 μmol/L).

### Neuroimaging and Electrophysiology

**Figure 1:**
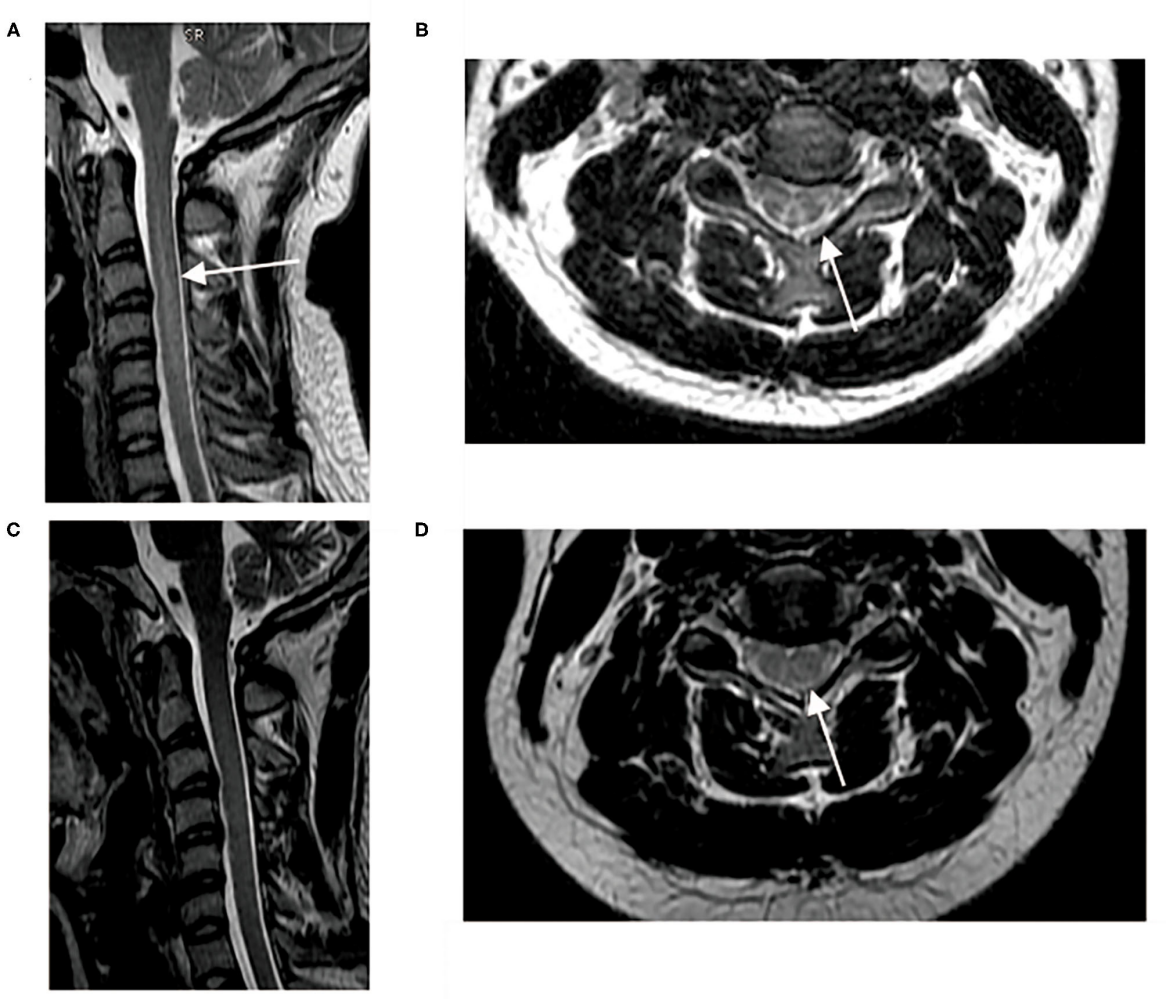
Spinal cord MRI pre- and after-treatment. **(A,B)** T2 hyperintensity of posterior column from C2–C6 pre-treatment; **(C)** Sagittal T2-hyperintensity of posterior column from C2–C6 resolved 4 months after treatment with mecobalamine and hyperbaric oxygen; **(D)** Axial inverted “V” T2-hyperintensity was blurred 4 months after treatment.

MRI of the spinal cord was performed in 19 patients. T2 hyperintensity of the posterior column in the spinal cord was found in 15 patients. The common segment involved started at C2 and terminated at C6 or C7. One patient (case 5) presented with myelopathy in the whole spinal cord. One patient (case 13) had several MRI scans of the spinal cord and changes in the MRI signal resolved after 4 months of treatment with mecobalamin and hyperbaric oxygen (Figure 1). It is noteworthy that, although abnormal spinal cord signals were seen in 15 patients, 13 patients presented with no sensory changes at the corresponding spinal level.

Eighteen patients underwent EMG, which showed demyelination and axonal damage in peripheral nerves in 16 patients. Twelve patients had both abnormal signals in the spinal cord and abnormal EMGs. The most common manifestations in our series were N_2_O-induced cervical myelopathy and peripheral neuropathy.

### Treatment and Prognosis

All patients ceased inhaling N2O and received supplements of cobamamide, mecobalamin or cyanocobalamin by intramuscular or intravenous injection (0.5–2 mg/day, Supplementary Table 2) for 13.7 ± 7.3 days, followed by oral medication. Five patients received glucocorticoid therapy, either to relieve edema of the spinal cord or because the patients did not admit to N_2_O abuse and a diagnosis of autoimmune disease was considered. Failure to admit to N_2_O abuse also resulted in two patients receiving IVIG. Five patients received hyperbaric oxygen therapy. Sixteen patients improved on discharge (Supplementary Table 2). After discharge, 16 patients recovered in 0.5–3.5 months, three patients were lost to follow-up and one patient still walked slowly and suffered from numb toes and memory loss.

### Literature Review

Data for 99 previously published patients with detailed medical records were collected (Table 1 and Supplementary Table 3). Men and women were almost equally represented, and the patients had a median age of 24.0 (Q1–Q3: 21.0–29.0) years old. Myelopathy was the most common presentation (42/99), followed by combined myelopathy and peripheral neuropathy (33/99) and peripheral neuropathy (11/99). Sixty-six patients presented with paresthesia or numbness as the initial symptoms, and 30 patients experienced gait instability. Over the course of the disease, 68 patients presented with weakness of the limbs, half of the lower limbs (31/69). During physical examination, 79 patients showed decreased deep sensory perception and 66 showed ataxia. Lhermitte's sign was rare (8/99). Reported abnormal laboratory data included elevated levels of homocysteine (93.2%) and MMA (94.1%), decreased levels of vitamin B12 (53.8%), elevated MCV (37.7%) and decreased Hb (31.3%). Spinal cord involvement was seen in 76 patients, most of which was in the cervical segment (52/76). Cervical and thoracic segments were involved in 20 patients. Only one patient had lesions in the thoracic segment. Notably, 50 patients did not present with changes in sensory perception at the corresponding spinal level, even though they had lesions in the spinal cord. Almost half of the patients had abnormal EMGs (45/99). Almost all the patients were treated with vitamin B12 (95/99). Most of the patients (73/99) improved following N_2_O cessation and vitamin B12 supplementation. One patient, whose medical history was concealed, deteriorated. One patient died because of re-abuse of N_2_O, combined with abuse of psychotropic drugs.

**Figure 2:**
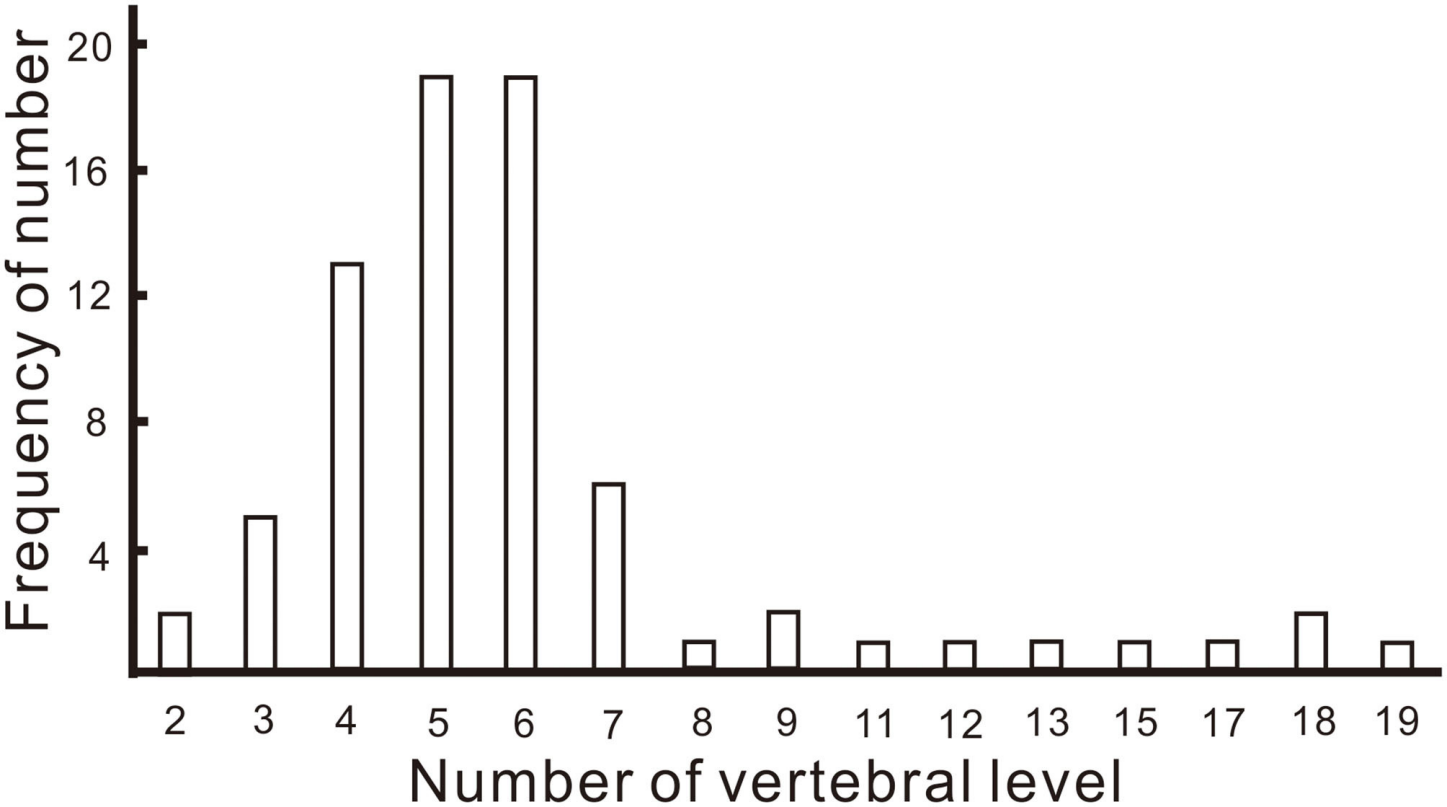
Frequency of the number of vertebral levels over which lesions of the spinal cord extended.

In addition, we analyzed the length of lesions in the spinal cord both in our own 20 patients and in 99 previously reported patients (Figure 2). In 25.3% of patients, the lesions extended over five or six vertebral levels, followed by four (17.3%), seven (8.0%) and three levels (6.7%).

### Subgroup Analysis

**Figure 3:**
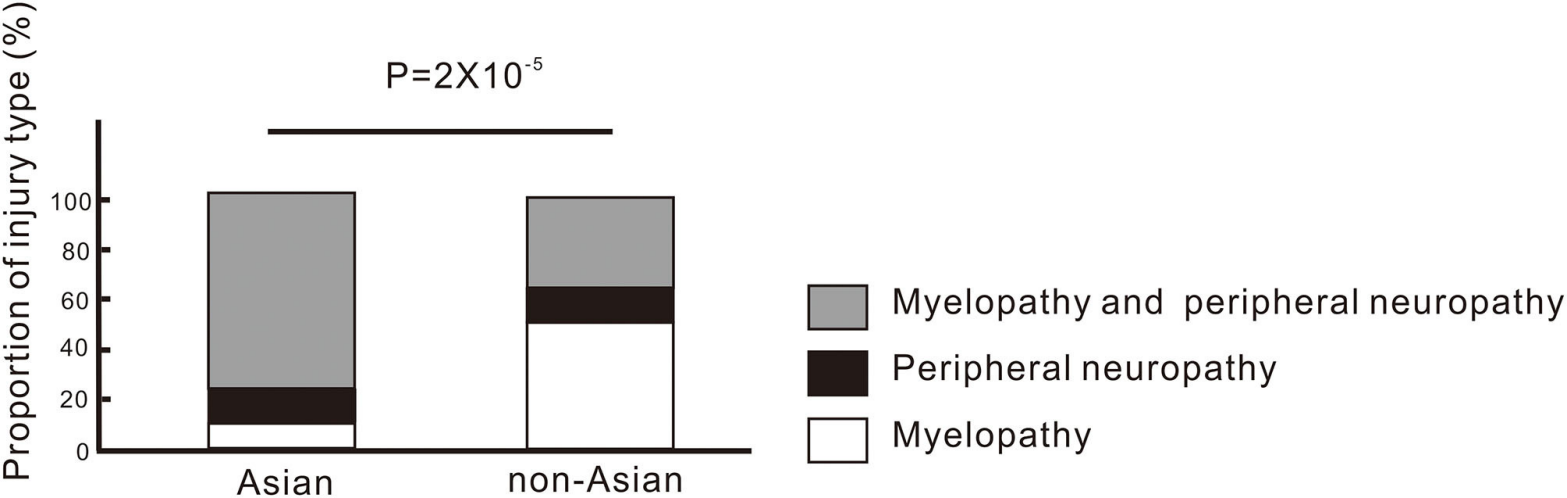
Type of injuries involved in N_2_O-induced neurological disorders in Asian and non-Asian patients.

The comparison between Asian patients and non-Asian patients is shown in Table 2. There were 56 Asian patients and 63 non-Asian patients. More Asian females abused N_2_O, whereas more non-Asian males abused N_2_O (χ^2^:8.868, *P* = 0.003). The Asian patients were significantly younger than non-Asian patients (*Z* = −3.632, *P* = 2.8 × 10^−4^). More non-Asian patients had a history of polysubstance or intravenous drug abuse (χ^2^: 5.521, *P* = 0.02). The average period of N_2_O abuse by Asian patients was significantly shorter than that of non-Asian patients (*Z* = −2.072, *P* = 0.04). Most Asian patients (41/56) had myelopathy combined with symptoms of, or EMG-confirmed peripheral injuries (combined injuries). Six patients had only myelopathy and seven had only peripheral injuries. Most non-Asian patients (26/63) had only myelopathy, followed by only peripheral injuries (7/63) and combined injuries (18/63). The proportions of the type of injury differed significantly between Asian and non-Asian patients (χ^2^: 21.398, *P* = 2 × 10^−5^, Figure 3 and Table 2). No significant differences in levels of Hb, MCV, vitamin B12 and homocysteine were found between Asian and non-Asian patients (*P* > 0.05). The comparison between mainland patients and Taiwan patients is shown in Table 3. No significant differences in the age, gender, the proportion of polysubstance or intravenous drug abuse, time of N_2_O abuse, levels of Hb, MCV, vitamin B12, homocysteine and the type of injuries were found between them.

**Table 2:** Comparison of demographic and clinical features of Asian and non-Asian patients.

		**Asian (56)**	**Non-Asian (63)**	***P*-value**
Age (years)		22.0 (19.0–26.0)	26.0 (22.3–31.0)	2.8 × 10^−4^
Sex (male/female)		23/33	43/20	0.003
Polydrug or intravenous drug abuse		4	14	0.02
Period of N_2_O abuse (months)		3.0 (2.0–10.5)	9.0 (2.0–24.0)	0.04
Vitamin B12(pg/ml)		156.0 (101.6–262.0)	160.0 (116.0–240.5)	1.0
Homocysteine(μmol/L)		44.1 (24.6–70.7)	51.7 (34.7–100.6)	0.2
Hemoglobin(g/L)		129.0 (104.0–141.0)	122.0 (106.0–142.0)	0.4
MCV(fl)		98.0 (93.5–101.9)	97.3 (94.1–100.0)	0.8
Type of injuries	Combination	41	18	2 × 10^−5^
	Myelopathy	6	26	
	Peripheral neuropathy	7	7	
	Unknown	2	12	

*Continuous variables were expressed as median (Q1–Q3); MCV, Mean Corpuscular Volume*.

**Table 3:** Comparison of demographic and clinical features of Chinese people in mainland and Taiwan.

		**Mainland (31)**	**Taiwan (12)**	***P*-value**
Age (years)		22.0 (19.0–24.0)	21.0 (19.0–25.8)	1
Sex (male/female)		15/16	4/8	0.4
Poly drug or intravenous drug abuse		1	2	0.1
Period of N_2_O abuse (months)		4.0 (2.0–9.0)	3.0 (2.0–24.0)	0.6
Vitamin B12(pg/ml)		147.0 (98.2–319.0)	211.8 (154.3–246.5)	0.3
Homocysteine(μmol/L)		38.8 (24.4–68.2)	33.3 (10.0–52.6)	0.3
Hemoglobin(g/L)		134.0 (101.0–145.5)	128.0 (115.0–144.5)	0.9
MCV(fl)		98.1 (92.0–102.3)	98.7 (95.9–101.7)	0.7
Type of injuries	Combination	20	12	0.07
	Myelopathy	4	0	
	Peripheral neuropathy	6	0	
	Unknown	0	0	

*Continuous variables were expressed as median (Q1–Q3); MCV, Mean Corpuscular Volume*.

## Discussion

In this study, we found that the most common initial symptoms of N_2_O-induced neurological disorders were weakness and/or paresthesia. Longitudinally extensive cervical myelopathy, with or without peripheral neuropathy, is the most common manifestation, but most patients with myelopathy had no sensory change at the corresponding spinal level. The most common number of vertebral levels involved in lesions was five and six. The most reliable and practical laboratory indicator for diagnosis was homocysteine. The Asian patients with N_2_O abuse were younger than non-Asian patients. The incidence of myelopathy combined with peripheral neuropathy was significantly higher in Asian patients than in non-Asian patients, who mainly had myelopathy or peripheral neuropathy.

One of the common manifestations of neurological disorders caused by N_2_O is SCD, which is characterized by decreased deep sensation, symmetrical T2-weighted hypersensitivity in the posterior column of spinal cord and vitamin B12 deficiency (9). However, attention must be given to the cause of differences between neurological disorders caused by N_2_O compared to those caused by vitamin B12 deficiency. In both our case series and previous cases, the cervical segment is much more vulnerable in patients who abuse N_2_O (3, 16). Zheng et al. found that 32 patients had lesions in the cervical spinal cord and one patient had lesions in both cervical and thoracic spinal cord in 33 patients with N_2_O abuse who performed MRI (3). Bao et al. found that, in 16 patients who abused N_2_O, the most commonly impaired vertebral levels were C3–C5 (50.6%), followed by C2 (41.6%) and C6 (37.2%) (16). Lan et al. suggested that the vulnerability of the cervical segment to N_2_O neurotoxicity is attributable to the higher density of myelinated fibers of fasciculus gracilis in the cervical segment compared with the thoracic segment (17, 18). In contrast, the upper thoracic segments of the spinal cord are affected in more patients with SCD secondary to vitamin B12 deficiency than in patients who abuse N_2_O (17, 19). In addition to the involvement of different segments of the spinal cord, differences in both nerve excitability and MRI T2 signals have been reported in N_2_O abuse and vitamin B12 deficiency (20). We also found that, in 25.3% of patients, the lesions in the spinal cord extended over five or six vertebral levels, followed by four levels. This means that the lesions in the spinal cord secondary to N_2_O abuse are longitudinally extensive myelitis. The extended vertebral levels in our study were a little longer than those in the study by Bao et al. (16) and similar to those in the study by Zheng et al. (3). An interesting observation is that, despite lesions in the cervical segment of the spinal cord, many patients had no sensory change at the corresponding spinal level. A low proportion of patients with sensory change at the spinal level was also seen by Bao et al. (16). Vasconcelos et al. considered that an absence of sensory change at the spinal level might be associated with better clinical resolution since the absence of sensory change at the spinal level indicated that transmission of sensory impulses to higher centers is preserved (18). Another explanation might be that dysfunction of the spinal cord is subclinical, although the lesions in the spinal cord seem extensive.

We found that, except for MMA levels, the biggest change in laboratory indicators was hyperhomocysteinemia, followed by levels of vitamin B12. Neither low Hb nor high MCV occurred frequently. Since N_2_O affects both methionine synthase and MMCoA mutase pathways, low Hb, elevated MCV, low vitamin B12 levels, high homocysteine and MMA levels, can be tested and helpful for differential diagnosis, although the specificity and sensibility of these indicators varies. Anemia was found in 66.2% of patients with SCD secondary to vitamin B12 deficiency in the study by Cao et al. (21). By contrast, anemia was found in only 15% of patients in our case series and 31.3% in previous cases. The low incidence of anemia in patients with symptoms secondary to N_2_O abuse indicates the involvement of mechanisms other than disturbed vitamin B12 metabolism, such as cytotoxicity of homocysteine toward cortical astrocytes and N_2_O-catalyzed production of hydroxyl radicals (22, 23). Low levels of vitamin B12 were seen in more patients than low levels of Hb. Theoretically, vitamin B12 should be a good indicator, but it is, on the contrary, a confusing indicator. On the one hand, some patients had taken vitamin B12 before hospitalization. On the other hand, in some patients, the serum vitamin B12 level is normal but with abnormal vitamin B12-related metabolism (functional vitamin B12 deficiency) (4). Both availability of folate and vitamin B12 deficiency affect levels of homocysteine, whereas levels of MMA are not affected by other vitamins (9). MMA is, therefore, an accurate and specific biomarker of functional vitamin B12 deficiency. Although the incidence of high levels of MMA in previous cases was 94.1%, only 34.3% of patients had an MMA test. This means that MMA is not a suitable indictor for practical use and the most sensitive and practical indicator for the diagnosis of N_2_O-induced neurological disorders is homocysteine.

In general, young people tend to abuse N_2_O for recreational purpose. Asian patients who abused N_2_O were younger than non-Asian patients. The Asian patients were mostly from China and Korea, where recreational N_2_O abuse is popular among young people (age range: 16–41). By contrast, in non-Asian countries, more middle-aged people abuse N_2_O, with even one 60-year-old women inhaling N_2_O (age range: 17–60) (24). The difference in age might be explained by different cultures and economies.

Our data showed that Asian patients were prone to combined injuries, whereas more non-Asian patients had single injury-lesions in the spinal cord. Neurological dysfunction secondary to N_2_O abuse is related to disturbed metabolism of vitamin B12, folate and homocysteine (9). *MTHFR* (methylenetetrahydrofolate reductase) is one of genes that participates in homocysteine metabolic pathways. Mutation of *MTHFR* attenuates the activity of MTHFR, resulting in hyperhomocysteinemia (15). The frequency of different genotypes of *MTHFR* differs among populations (15), and the strength of the association between *MTHFR* and risk of coronary heart disease was found to vary in eastern and western populations (25). The differences in types of injury seen in neurological disorders caused by N_2_O abuse in different populations may, therefore, be explained by differences in genetic background. Additionally, Asian people eat less meat than European or North American people, which may influence basal serum levels of vitamin B12 since dietary vitamin B12 is mainly derived from animal sources (26). It has been reported that symptoms secondary to N_2_O abuse may be related to basal serum levels of vitamin B12 and the difference may, therefore, also be associated with different dietary habits (27). Our data also showed that there were no significant differences in demographical and clinical characteristics of N_2_O abuse between mainland patients and Taiwan patients. The results of mainland patients were almost parallel with that of the previous study on Taiwan patients, except for N_2_O exposure time, proportion of multiple illicit substance abuse, and serum vitamin B12 (28). These differences may be attributed to social factors and vitamin B12 supplement before hospitalization.

N_2_O-induced neurological disorders are mostly regarded as SCD, but there are some differences between the two. If longitudinally extensive cervical myelopathy with peripheral neuropathy is found in young patients with a normal diet, clinicians should consider the possibility of N_2_O abuse in either Neurology or Emergency departments, regardless of presence of amenia or low serum vitamin B12. The present study has some limitations. Firstly, the sample size in the retrospective study was small. We plan to conduct a large sample study with follow-up. Secondly, not all of necessary laboratory tests and ancillary examinations were performed on all of our patients. This means that there are some gaps in our results, which influence our interpretation. Lastly, since not all of our patients were followed-up regularly, and follow-up consisted mainly of telephone interviews, dynamic changes in symptoms, MRI and EMG were not observed regularly.

## Conclusions

N_2_O abuse is increasing rapidly among young people in a number of countries. It is very important that all frontline clinicians are aware of the damage caused by N_2_O, and of the various clinical presentations of neurotoxicity related to abuse of N_2_O. The key clinical manifestations associated with N_2_O abuse are longitudinally extensive cervical myelopathy and peripheral neuropathy in young patients, which should enable a quick and accurate diagnosis. The type of injury differs in Asian patients and non-Asian patients, which makes differential diagnosis more difficult.

## Data Availability Statement

The original contributions presented in the study are included in the article/Supplementary Material. Further inquiries can be directed to the corresponding authors.

## Ethics Statement

The studies involving human participants were reviewed and approved by Clinical Research Ethics Committee of Anhui Medical University. The patients/participants provided their written informed consent to participate in this study.

## Author Contributions

JZ, YT, and KW contributed to the study design. JZ, DX, XY, CW, and XL contributed to the data collection. JZ, YZ, YJ, YT, and NZ contributed to the data analysis and interpretation. JZ, YT, YJ, and XJ contributed to the manuscript preparation. All authors contributed to the article and approved the submitted version.

## Conflict of Interest

The authors declare that the research was conducted in the absence of any commercial or financial relationships that could be construed as a potential conflict of interest.
