# Supplementary material for: Key Characteristics of Nitrous Oxide-Induced Neurological Disorders and Differences Between Populations
Source: Front Neurol. 2021 Apr 27;12:627183. doi: 10.3389/fneur.2021.627183 (PMC8110825; doi:10.3389/fneur.2021.627183)
Supplement: Supplementary file 1 [file Data_Sheet_1.pdf]

Supplementary Table 1 Clinical features of 20 patients in current cohort

No.	Age（Y）/Sex	Period of N_2O abuse	Complaint	Motor	Sensory	Ataxia	Other symptoms	Vitamin B12 (pg/ml)	Hb (g/L)	MCV (fl)	HCY (μmol/L)	Spinal cord MRI	EMG
1	16/F	1M	Weakness and numbness of four limbs and unsteady walk for 4 days	Four limbs (4/5)	Decreased pinprick sensation	No	No	147	104	104.3	3.4	C2-C6（SCD）	Demyelination
2	18/F	1Y	Progressive weakness and numbness of four limbs and unsteady walk for 8 months	Distal parts of four limbs（5-/5）	Decreased pinprick sensation below the elbows,increased pinprick sensation of dorsum feet	Yes	No	↑	151	84.0	/	/	Demyelination
3	19/F	0.5M	Numbness of four limbs for half a month	Distal part of lower limbs (4/5),hyporeflexia	Decreased pinprick sensation below the ankles and wrists, decreased deep sensation of lower limbs	Yes	No	161	83	67.9	20.3	C3-C6 (SCD)	Axonal damage and demyelination
4	20/F	4M	Numbness of four limbs for 2 months, weakness of four limbs for 4 days	Four limbs （4/5),absent tendon reflexes	N	No	No	N	N	N	43	C2-C7（SCD）	Axonal damage and demyelination
5	22/F	4M	Weakness of lower limbs and unsteday walk for 1 week	Lower limbs (4/5)	Abnormal deep sensory	Yes	No	/	98	112.2	24.7	Abnormal signal in the whole spinal cord	/
6	22/F	3D	Numbness and weakness of upper limbs for 10 days	Distal parts of four limbs (5-/5),hypomyotonia, hyporeflexia, postural tremor of upper limbs	Decreased pinprick sensation below the elbows,decreased motor sensation of toes	Yes	Irritability, bad sleep quality	/	120	92.2	127.2	N	N
7	22/F	1M	Weakness and numbness of four limbs for 1 week	Distal parts of lower limbs(4/5),hyporeflexia	Decreased pinprick sensation below the elbows,decreased motor sensation of toes	Yes	No	136	118	98.8	16.8	C1-T2	Axonal damage and demyelination

No.	Age（Y）/Sex	Period	Complaint	Motor	Sensory	Ataxia	Other symptoms	Vitamin B12 (pg/ml)	Hb (g/L)	MCV (fl)	HCY (μmol/L)	Spinal cord MRI	EMG
8	24/F	2M	Weakness and numbness of four limbs and unsteady walk for 1 week	Four limbs(upper:5-/5,lower:3/5), hypomyotonia	Decreased pinprick sensation	No	No	319	140	98.1	20.6	C2-C6（SCD）	Axonal damage and demyelination
9	24/F	1Y	Weakness of lower limbs for half of year	Weakness of lower limbs(4/5),hyporeflexia	Paroxysmal nubmness of distal parts of all limbs, but sensation examation is normal	No	No	91	126	102.8	66.17	N	Axonal damage and demyelination
10	17/M	3M	Progressive weakness and numbness of four limbs	Weakness of four limbs(4/5), hypomyotonia, hyporeflexia	Decreased pinprick sensation below the elbows and knee joints	Yes	No	/	149	92.8	91.3	C2-C5 (SCD)	Demyelination
11	21/M	2Y	Weakness and numbness of lower limbs for 1 week	Decreased muscle strength of dorsal stretch of feet	Decreased deep sensation	Yes	No	92.31	157	90.2	102	C3-C6 (SCD)	/
12	22/M	6M	Numbness of four limbs for 1 week	Four limbs(5-/5),hypotonia, hyporeflexia of lower limbs	Decreased pinprick sensation of distal parts of four limbs,decreased deep sensation of lower limbs	Yes	Hallucination	/	138	102.7	35.72	C1-C7 (SCD)	Axonal damage
13	23/M	6M	Weakness of lower limbs for 10 days and numbness of four limbs for 2 days	Four limbs(5-/5),decreased muscle strength of dorsal stretch of feet,hyporeflexia	Decreased touch sensation and hyperalgesia of distal parts of four limbs,decreased deep sensation of lower extremities	No	No	>2000	134	103.6	7.92	C2-C6（SCD）	Axonal damage and demyelination
14	24/M	2M	Weakness of lower limbs for 2 months, worse for 2 days	Lower limbs(3/5), bilateral babinski signs (+)	N	No	No	224	133	94.6	24.4	C2-C7（SCD）	Demyelination
15	27/M	3M	Weakness and numbness of four limbs for 3 months	Hyperreflexia of lower limbs	Decreased pinprick sensation of distal parts of four limbs	No	Root pain,dyspnea,palpitation,impaired memory	975	154	97.2	64.65	N	Axonal damage and demyelination
16	28/M	1Y	Parosymal numbness of four limbs for 2 months	N	Decreased pinprick sensation	No	No	/	150	90.2	33.9	C2-C6（SCD）	Axonal damage and demyelination

No.	Age（Y）/Sex	Period	Complaint	Motor	Sensory	Ataxia	Other symptoms	Vitamin B12 (pg/ml)	Hb (g/L)	MCV (fl)	HCY (μmol/L)	Spinal cord MRI	EMG
17	29/M	6M	Numbness of four limbs for 1 months	Hyporeflexia of upper limbs,left hoffman(+)	Decreased pinprick sensation from C3-T6	No	No	978	148	91.8	/	C2-C5（SCD）	N
18	31/M	6M	Weakness and numbness of the whole body for 2 weeks	Four limbs (5-/5),unsteady walk,hyporeflexia	Decreased pinprick sensory	Yes	Bowel and bladder dysfunction	1191	139	95.8	/	Protrusion of cervical intervertebral disc	Axonal damage and demyelination
19	32/M	2M	Numbness of four limbs for 3 days	Lower limbs(4/5）	Decreased pain sensation below groin and below wrist joints	Yes	No	/	141	98.2	68.2	C2-C5 (SCD)	Axonal damage
20	35/M	1Y	Numbness of left leg for 2 days	Amyotrophy of dorsum of left foot	Decreased pinprick and deep sensation	Yes	Artery thrombosis in lower limbs,aorta abdominalis,pain	/	137	98.8	/	C2-Th7 (SCD)	Axonal damage and demyelination

Muscle strength assessments according to UK Medical Research Council (MRC);Motor: including MBC, muscle tone and tendon reflex;Hb:hemoglobin;MCV:Mean Corpuscular Volume;HCY:homocysteine;;EMG:Electromyography;/:unknown; N:normal; ↑:increase;Y:years;M;month;D:day;SCD:Subacute combined degeneration of spinal cord;IVIG:intravenous immunoglobulin;Normal range: vitamin B12:197–771 pg/mL; Hb: female >110 g/L; male >120 g/L; MCV:<100 fl; HCY:5–13.9 μmol/L.

Supplemenatary Table 2 Treatment and prognosis of 20 patients in current cohort

No.	In hospital treatment	Symptoms and physical signs when discharged	Post-discharge treatment	Time of complete remission after discharge	Remarks
1	Cobamamide 0.5mg/D*17D, glucocorticoid	Normal muscle strength, improved sensation	Mecobalamine 1.5mg/D*3M	3M	Relapse of N_2O
2	Mecobalamine 2mg/D*16D, IVIG	Improved muscle strength of lower limbs	Mecobalamine 1.5mg/D*3M	3M	
3	Cobamamide 0.5mg/D*17D， mecobalamine 0.5mg/D*9D	Improved sensation	Mecobalamine 1.5mg/D	Loss to follow-up	
4	Mecobalamine 0.5mg/D*10D, glucocorticoid	Improved muscle strength of four limbs	Mecobalamine 0.5mg/D	Loss to follow-up	
5	Vitamin B12 1.0mg/D*14D	Improved muscle strength of lower limbs	Cobamamide 2.25mg/D*1M	0.5M	
6	Mecobalamine 2mg/D*7D	Improved sensation of upper limbs, normal sleep	Mecobalamine 1.5mg/D*3M	3M	
7	Mecobalamine 0.5mg/D*16D	Improved muscle strength	Mecobalamine 1.5mg/D*1M	1M	
8	Cobamamide 0.5mg/D*14D, glucocorticoid	Improved muscle strength of four limbs	Mecobalamine 1.5mg/D*2M	2M	
9	Mecobalamine 2mg/D*7D, glucocorticoid	Improved muscle strength of four limbs	No vitamin	3M	
10	Mecobalamine 2mg/D*14D, mecobalamine 1mg/D*14D	Improved muscle strength of four limbs,normal EMG	Mecobalamine 1.5mg/D*3M	3M	
11	Vitamin B12 1.0mg/D*1M	Improved muscle strength of lower limbs	Vitamin B12 1mg/D*10D, cobamamide 2.25mg/D*2M	2M	
12	Mecobalamine 0.5mg/D*8D, hyperbaric oxygen	Improved sensation	Mecobalamine 1.5mg/D*2M， hyperbaric oxygen	0.5M	Relapse of N_2O
13	Mecobalamine 0.5mg/D*7D, cobamamide 1.5mg/D*7D， hyperbaric oxygen, IVIG	Improved muscle strength and sensation	Mecobalamine 1.5mg/D*3M	3M	
14	Cobamamide 0.5mg/D*8D	No obivous improvement	Mecobalamine 1.5mg/D*2M	2M	
15	Mecobalamine 1mg/D*8D, hyperbaric oxygen	Improved muscle strength and sensation	Mecobalamine 1.5mg/D*5M	5M	Relapse of N_2O
16	Cobamamide 0.5mg/D*4D	No improvement	Mecobalamine 1.5mg/D*1M	1M	
17	Cobamamide 0.5mg/D*15D， glucocorticoid	No obivous improvement	Mecobalamine 1.5mg/D*0.5M	0.5M	
18	Cobamamide 0.5mg/D*10D, hyperbaric oxygen	Improved muscle strength	Mecobalamine 1.5mg/D*3.5M	3.5M	
19	Cobamamide 0.5mg/D*6D, hyperbaric oxygen	Exacerbate numbness	Mecobalamine 1.5mg/D*6M	Walking slowly, numb toes,memory loss*	
20	Vitamin B12 1.0mg/D*3D, mecobalamine 0.5mg/D*13D	Improved sensation	Mecobalamine 1.5mg/D	Loss to follow-up	

Only the dosage of vitamin B12 is listed because vitamin B12 is the most importment treatment for the patients ; M:month;D:day;IVIG:intravenous immunoglobulin; *: the patient still had some symptoms 16 months after discharge

Supplementary Table 3 Clinical data of 99 patients reported previously

No.	Age/ Sex	Clinical manifestation	Laboratory test	Spinal cord imaging	EMG	Treatment	Prognosis	Citation
1	28/M	Paresthesia,weakness,decreased deep senstaion	VitB12↓,HCY↑,Hb,MCV:N,MMA↑	C4-C6	A	VitB12, IVIG	Improvement	Algahtani et al.2020
2	22/M	Paresthesia,weakness,decreased deep senstaion,ataxia,bowel or bladder dysfunction	VitB12:N,HCY↑,Hb↓,MCV↑,MMA↑	C1-C6	/	VitB12	Improvement	Al-Sadawi et al.2018
3	24/M	Paresthesia,decreased deep senstaion,involuntary movement,impaired cognition,pain	VitB12,Hb,MCV:N,HCY↑,MMA↑	/	/	VitB12	Lost	Alt et al.2011
4	29/F	Paresthesia,weakness,decreased deep senstaion,ataxia	VitB12↓,Hb↓,MCV:N,MMA↑	Cervical and thoracic	/	VitB12	Lost	Anderson et al.2018
5	60/M	Ataxia,psychiatric symptoms	VitB12↓,HCY↑,MMA↑	C3-C5	/	VitB12	Improvement	Anderson et al.2015
6	25/M	Paresthesia,decreased deep senstaion,ataxia,lhermitte symptom	VitB12:N,HCY↑,MMA↑	C1-C6	/	VitB12	Improvement	Antezana et al.2015
7	20/M	Paresthesia,weakness,decreased deep senstaion	VitB12↓,MCV↑	C2-C4	/	VitB12	Improvement	Antonucci et al.2018
8	21/M	Paresthesia,decreased deep senstaion,ataxia	VitB12:N,HCY↑	C2-C7	/	VitB12	Improvement	Arshi et al.2014
9	27/F	Paresthesia,weakness,decreased deep senstaion,ataxia	VitB12:N,HCY↑	C2-C6	/	VitB12	Improvement	Brosset et al.2020
10	31/M	Paresthesia,weakness,decreased deep senstaion	VitB12:N,MMA↑	C1-C6	/	VitB12, steroid	Improvement	Buizert et al.2017
11	23/M	Decreased deep senstaion,ataxia	VitB12,Hb,MCV:N	C1-C6	N	VitB12, methionine	Improvement	Butzkueven et al.2000
12	42/F	Paresthesia,decreased deep senstaion,ataxia, impaired cognition,pain	VitB12↓,HCY↑,Hb,MCV:N,MMA↑	C4-C5	/	VitB12	Improvement	Campdesuner et al.2020
13	20/F	Weakness, decreased deep senstaion,psychiatric symptoms,thrombosis	VitB12↓,Hb↓,MCV:N	Abnormal	/	VitB12, methionine	Improvement	Cartner et al.2007
14	20/F	Paresthesia,decreased deep senstaion,involuntary movement	Hb:N,MCV:N	C1-C6	A & D	VitB12	Improvement	Chen et al.2016
15	Na/F	Paresthesia,weakness,decreased deep senstaion,ataxia	VitB12↓	C2-C5	/	VitB12	/	Cheng et al.2013
16	29/F	Paresthesia,weakness,decreased deep senstaion,ataxia,bowel or bladder dysfunction,hyperpigmentation	VitB12↓,HCY↑,Hb↓,MCV:N	/	/	VitB12	Improvement	Chiang et al.2013
17	24/M	Paresthesia,decreased deep senstaion,ataxia,bowel or bladder dysfunction	VitB12, Hb:N,HCY↑,MCV↑	C1-C7	D	VitB12	Improvement	Choi et al.2019
18	22/F	Paresthesia,weakness,decreased deep senstaion,ataxia,bowel or bladder dysfunction	VitB12↓,HCY↑,Hb,MCV:N	C3-C5	A	VitB12	Improvement	Choi et al.2019
19	37/M	Paresthesia,decreased deep senstaion,ataxia	VitB12,Hb:N,HCY↑,MCV↑,MMA↑	C3-C5	/	VitB12	Remain	Chomin et al.2018
20	24/M	Paresthesia,ataxia	VitB12,Hb,MCV:N,HCY↑	C2-C6	/	VitB12	Improvement	Demas et al.2020
21	31/M	Paresthesia,weakness,decreased deep senstaion,ataxia,involuntary movement	VitB12↓,HCY↑ Hb,MCV:N	Cervical and thoracic	/	VitB12	Improvement	Diamond et al.2004
22	22/M	Paresthesia,weakness,decreased deep senstaion, bowel or bladder dysfunction	VitB12↓,HCY↑,Hb,MCV:N	C1-C6	A & D	VitB12	Improvement	Dong et al.2019
23	20/F	Paresthesia,weakness,decreased deep senstaion,pain	VitB12↓,Hb,MCV:N,MMA↑	Abnormal	D	VitB12, steroid, IVIG	Improvement	Duque et al.2015
24	27/F	Paresthesia,weakness,decreased deep senstaion,ataxia	VitB12↓,HCY↑,Hb↓,MCV↑,MMA↑	/	/	VitB12	Improvement	Edigin et al.2019

No.	Age/ Sex	Clinical manifestation	Laboratory test	Spinal cord imaging	EMG	Treatment	Prognosis	Citation
25	24/F	Ataxia,pain,hyperpigmentation	VitB12↓,HCY↑,Hb:N,MCV↑,MMA↑	C2-C7	/	VitB12	/	Egan et al.2018
26	30/M	Paresthesia,weakness,decreased deep senstaion,ataxia	VitB12:N,MMA↑	C1-C6	/	VitB12	Improvement	Ernst et al.2015
27	17/F	Paresthesia,weakness,decreased deep senstaion,hyperpigmentation	VitB12↓,HCY↑,Hb↓,MCV:N	C2-C5	A&D	VitB12	Improvement	Fang et al.2020
28	19/F	Paresthesia,weakness,decreased deep senstaion,hyperpigmentation	VitB12↓,HCY↑,Hb↓,MCV:N	C2-C5	A&D	VitB12	Improvement	Fang et al.2020
29	23/M	Paresthesia,weakness,decreased deep senstaion,hyperpigmentation	VitB12↓,HCY↑,Hb↓,MCV↑	N	A	VitB12	Improvement	Fang et al.2020
30	20/F	Paresthesia,hyperpigmentation	VitB12↓,HCY↑Hb,MCV:N	N	D	VitB12	Improvement	Fang et al.2020
31	27/M	Paresthesia,weakness,decreased deep senstaion,ataxia,pain	VitB12↓,HCY↑,Hb,MCV:N	Cervical and thoracic	/	VitB12	Improvement	Fernandez et al.2017
32	22/F	Paresthesia,decreased deep senstaion,ataxia	VitB12:N,HCY↑	C2-C6	D	VitB12	Improvement	Fong et al.2016
33	38/M	Paresthesia,decreased deep senstaion,ataxia,psychiatric symptoms	VitB12↓,HCY↑,Hb↓,MCV↑,MMA↑	/	/	VitB12	Improvement	Forzani et al.2013
34	24/F	Paresthesia,weakness	VitB12,Hb,MCV:N,HCY↑,MMA↑	Abnormal	/	VitB12	/	Francis et al.2019
35	19/M	Paresthesia,weakness,decreased deep senstaion,ataxia,pain	VitB12:N	C2-C7	/	Steroid	Improvement	Ghobrial et al.2012
36	23/F	Paresthesia,weakness,decreased deep senstaion,thrombosis	VitB12↓,HCY↑,Hb↓,MCV:N,MMA↑	N	A&D	VitB12	Improvement	Glijn et al.2017
37	45/M	Paresthesia,weakness,decreased deep senstaion,ataxia,psychiatric symptoms , impaired cognition,pain	VitB12↓,HCY↑,Hb,MCV:N	/	A	VitB12	Improvement	Hew et al.2018
38	23/M	Paresthesia,weakness,decreased deep senstaion,ataxia	VitB12↓,HCY↑	N	A&D	VitB12	Death	Hirvioja et al.2016
39	19/M	Paresthesia,weakness,ataxia,involuntary movement	VitB12↓,Hb:N,MCV↑	C1-C5	D	VitB12	Improvement	Hsu et al.2012
40	16/F	Paresthesia,weakness,decreased deep senstaion,ataxia	VitB12,HCY,Hb,MCV:N	C1-C6,T7-T8	Abnormal	VitB12	Improvement	Hu et al.2014
41	19/F	Weakness,ataxia	/	Cervical and thoracic	D	/	/	Huang et al.2009
42	55/M	Decreased deep senstaion,psychiatric symptoms	VitB12↓,Hb:N,MCV↑	Abnormal	/	/	Improvement	Iwata et al.2001
43	19/F	Paresthesia,weakness,decreased deep senstaion,ataxia	VitB12:N,HCY↑,MCV↑	C2-C6	A & D	VitB12, steroid	Improvement	Ji et al.2018
44	24/M	Paresthesia,weakness,decreased deep senstaion,ataxia,lhermitte symptom	VitB12↓,HCY↑,Hb↓	C2-C6	/	VitB12	Improvement	Jiang et al.2020
45	21/F	Weakness,decreased deep senstaion,ataxia,impaired cognition,hyperpigmentation	VitB12↓,Hb↓	C2-C7	A & D	VitB12	Improvement	Johnson et al.2018
46	18/F	Paresthesia,weakness,ataxia	VitB12,MMA:N	Cervical and thoracic	/	VitB12	Improvement	Jones et al.2015
47	19/F	Paresthesia,decreased deep senstaion,ataxia	VitB12↓,HCY↑,Hb↓,MCV↑,MMA↑	C1-T4	/	VitB12	Improvement	Kanin et al.2020
48	27/M	Paresthesia,decreased deep senstaion	VitB12,Hb,MCV:N,HCY↑,MMA↑	N	A	VitB12	Improvement	Kaski et al.2017

No.	Age/ Sex	Clinical manifestation	Laboratory test	Spinal cord imaging	EMG	Treatment	Prognosis	Citation
49	22/F	Paresthesia,weakness,decreased deep senstaion,ataxia,thrombosis	VitB12↓,MCV↑	C1-C5	A	VitB12	Improvement	Kwon et al.2019
50	33/M	Paresthesia,weakness,decreased deep senstaion,ataxia	VitB12↓,MCV↑	C2-C6	A	VitB12	Improvement	Kwon et al.2019
51	32/F	Decreased deep senstaion,ataxia	VitB12↓,HCY↑,Hb:N,MCV↑	C1-2	/	VitB12	Improvement	Lee et al.2019
52	41/M	Paresthesia,weakness,decreased deep senstaion,ataxia,lhermitte symptom	VitB12↓,Hb:N,MCV↑	C2-C7	A	/	/	Lin et al.2007
53	24/M	Paresthesia,decreased deep senstaion,ataxia,bowel or bladder dysfunction	VitB12↓,Hb:N,MCV↑	C2-C6	A & D	VitB12	Improvement	Lin et al.2011
54	18/F	Paresthesia,weakness,decreased deep senstaion,ataxia,bowel or bladder dysfunction	VitB12,Hb:N,HCY↑,MCV↑	C2-C6	A & D	VitB12	Lost	Lin et al.2011
55	20/F	Paresthesia,weakness,decreased deep senstaion	VitB12,Hb:N,MCV↑	C2-C7	D	VitB12	Lost	Lin et al.2011
56	21/F	Weakness,ataxia,psychiatric symptoms	VitB12↓,HCY↑,Hb↓,MCV:N,MMA↑	N	/	VitB12	Improvement	Lundin et al.2019
57	35/M	Paresthesia,weakness,decreased deep senstaion,ataxia,psychiatric symptoms	VitB12↓,HCY↑,MMA↑	C2-C6	N	VitB12, methionine	Improvement	Mancke et al.2016
58	36/M	Paresthesia,decreased deep senstaion,ataxia, involuntary movement	VitB12↓,HCY↑,MCV↑	C1-Th11	A & D	VitB12	Improvement	Massey et al.2016
59	24/F	Paresthesia,weakness,decreased deep senstaion,ataxia	VitB12↓,HCY↑,Hb,MCV:N	C1-C5	A	VitB12	/	McArdle et al.2020
60	19/F	Weakness,ataxia,impaired cognition	VitB12↓,Hb↓,MCV:N	C3-C6	A	VitB12	/	McArdle et al.2020
61	19/F	Paresthesia,ataxia,psychiatric symptoms ,bowel or bladder dysfunction	VitB12↓,MCV↑	C1-C5	A	VitB12	/	McArdle et al.2020
62	18/F	Paresthesia,weakness,ataxia	VitB12↓,Hb,MCV:N	C1-thoracic	A	VitB12	/	McArdle et al.2020
63	22/M	Paresthesia,weakness,thrombosis	VitB12:N	/	D	VitB12	Improvement	Middleton et al.2018
64	39/M	Decreased deep senstaion,ataxia	VitB12:N	/	/	VitB12	Improvement	Miller et al.2004
65	22/M	Paresthesia,weakness,decreased deep senstaion,ataxia,lhermitte symptom	VitB12↓,HCY↑,Hb:N,MCV↑	N	Abnormal	VitB12	Improvement	Morris et al.2015
66	24/M	Paresthesia,decreased deep senstaion,ataxia,involuntary movement	HCY↑,MMA↑	N	/	VitB12	Lost	Morrissey et al.2009
67	37/M	Paresthesia,weakness,ataxia,psychiatric symptoms ,bowel or bladder dysfunction	VitB12↓,Hb,MCV:N	C1-C7	/	VitB12	Improvement	Ng et al.2002
68	30/M	Paresthesia,decreased deep senstaion,ataxia,thrombosis	VitB12:N,HCY↑,Hb↓,MMA↑	N	/	VitB12	Improvement	Olney et al.2019
69	30/F	Paresthesia,weakness,decreased deep senstaion,ataxia,lhermitte symptom	VitB12↓,HCY↑,Hb,MCV:N,MMA↑	C2-C7	/	VitB12	Improvement	Onrust et al.2019
70	Na/M	Weakness,decreased deep senstaion,ataxia	VitB12↓	Cervical	/	VitB12	/	Papathanasiou et al.2015
71	Na/M	Weakness,decreased deep senstaion,ataxia	VitB12:N	Cervical	/	VitB12	/	Papathanasiou et al.2015

No.	Age/ Sex	Clinical manifestation	Laboratory test	Spinal cord imaging	EMG	Treatment	Prognosis	Citation
72	31/M	Paresthesia,weakness,decreased deep senstaion,ataxia,Lhermitte symptom	VitB12↓,MCV↑	C2-C5	/	VitB12	Improvement	Pema et al.1998
73	31/F	Paresthesia,weakness,decreased deep senstaion	/	C1-T8	/	VitB12	Lost	Prigge et al.2012
74	27/F	Paresthesia,weakness,decreased deep senstaion,ataxia,bowel or bladder dysfunction,pain	VitB12,HCY,MCV:N,Hb ↓,MMA↑	C3/C4-T11/T12	/	VitB12	Lost	Pugliese et al.2015
75	35/M	Paresthesia,weakness,ataxia,impaired cognition,bowel or bladder dysfunction	VitB12,Hb,MCV:N	C2-C7	/	VitB12	Remain	Rheinboldt et al.2014
76	28/M	Paresthesia,decreased deep senstaion	VitB12,Hb,MCV:N,HCY↑	/	/	VitB12	Remain	Richardson et al.2010
77	25/M	Paresthesia,ataxia	VitB12,Hb:N	Cervical and upper thoracic	/	VitB12, methionine	Improvement	Sadr et al.2010
78	22/M	Paresthesia,weakness,decreased deep senstaion,ataxia	VitB12,Hb,MCV:N，HCY ↑,MMA↑	C2-C5	/	VitB12	Improvement	Seed et al.2020
79	45/M	Paresthesia,weakness,decreased deep senstaion,ataxia	VitB12↓,HCY ↑,Hb,MCV:N,MMA↑	Cervical and thoracic	/	VitB12	Improvement	Shah et al.2019
80	22/F	Paresthesia,weakness,decreased deep senstaion,impaired cognition,bowel or bladder dysfunction	VitB12,MCV:N,HCY↑	C1-T11	Abnormal	VitB12	Improvement	Shen et al.2019
81	23/F	Paresthesia,weakness,bowel or bladder dysfunction,thrombosis	Hb↓,MCV:N	C1-T12	A	VitB12	Improvement	Shulman et al.2007
82	28/M	Paresthesia,decreased deep senstaion	VitB12↓	Cervical	/	VitB12,IVIG	Improvement	Simpson et al.2019
83	29/F	Paresthesia,weakness,decreased deep senstaion,bowel or bladder dysfunction	VitB12:N	C2-T5	/	VitB12	Improvement	Sleeman et al.2016
84	28/M	Paresthesia,decreased deep senstaion,bowel or bladder dysfunction, lhermitte symptom	VitB12,Hb:N	Caudal medulla to C7	/	VitB12	Improvement	Sotirchos et al.2012
85	25/F	Paresthesia,ataxia	VitB12:N,HCY↑	N	Abnormal	VitB12, steroid	Improvement	Spickler et al.2019
86	22/M	Paresthesia,weakness,decreased deep senstaion,ataxia	VitB12,MCV:N,HCY↑,Hb ↓,MMA↑	C1-T5	/	VitB12	Improvement	Stockton et al.2017
87	46/F	Paresthesia,weakness,decreased deep senstaion,impaired cognition	VitB12:N,HCY↑,Hb↓,MCV ↑,MMA↑	N	/	VitB12	Improvement	Sutaria et al0.2018
88	25/M	Paresthesia,weakness,decreased deep senstaion	VitB12:N	C2-C6	A & D	VitB12, steroid	Improvement	Tatum et al.2010
89	22/M	Paresthesia,weakness,decreased deep senstaion,ataxia	VitB12,Hb,MCV,MMA↑	N	D	VitB12, IVIG	Improvement	Thompson et al.2015
90	27/M	Paresthesia,weakness	VitB12,HCY,Hb,MCV,MMA: N	N	A & D	VitB12,IVIG	Worse	Thompson et al.2015
91	23/F	Paresthesia,weakness,decreased deep senstaion,ataxia,bowel or bladder dysfunction, lhermitte symptom,pain	VitB12:N,HCY↑	N	N	VitB12	Improvement	Thompson et al.2015
92	23/M	Paresthesia,decreased deep senstaion	VitB12↓,HCY↑,Hb:N,MCV ↑,MMA↑	/	/	VitB12	Improvement	Waclawik et al.2003

No.	Age/ Sex	Clinical manifestation	Laboratory test	Spinal cord imaging	EMG	Treatment	Prognosis	Citation
93	24/M	Paresthesia,decreased deep senstaion、ataxia,impaired cognition	VitB12↓,HCY ↑,MCV:N,MMA↑	C2-C7	/	VitB12	Remain	Waters et al.2005
94	25/M	Paresthesia,weakness,bowel or bladder dysfunction	/	C2-T1	Abnor mal	VitB12, steroid	Improvement	Weng et al.2017
95	17/M	Paresthesia,weakness,decreased deep senstaion,involuntary movement,bowel or bladder dysfunction	VitB12,Hb,MCV:N,HCY ↑,MMA↑	C1-C6/7	/	VitB12, steroid	Improvement	Williamson et al.2019
96	26/F	Paresthesia,weakness,decreased deep senstaion	VitB12↓,Hb:N,MCV↑	C2-C7	D	VitB12, plasma exchange	Improvement	Wu et al.2007
97	20/F	Paresthesia,weakness,decreased deep senstaion,ataxia,impaired cognition	VitB12,HCY,Hb,MCV:N	C1-T2	D	VitB12	Improvement	Yuan et al.2017
98	21/M	Paresthesia,weakness,decreased deep senstaion,ataxia	VitB12,Hb,MCV:N、HCY↑	C2-C6	D	VitB12	Improvement	Zhao et al.2020
99	18/F	Paresthesia,weakness,decreased deep senstaion	VitB12,MCV:N、HCY↑,Hb ↓	T3-T6	A&D	VitB12, high pressure oxygen	Improvement	Zhao et al.2020

Age: the unit is years old; Na: age which were not mentioned in literature; M: male; F: female; VitB12: Vitamin B12; HCY: homocysteine; Hb: hemoglobin; MCV: Mean corpuscular volume; MMA: methylmalonic acid; N: normal; ↑:increase; ↓： decrease; /:data which were not mentioned in literature; EMG: Electromyography; A: axonal; D: demyelination; IVIG: intravenous immunoglobulin

References

1.Algahtani H, Shirah B, Abdelghaffar N, et al. Nitrous oxide recreational abuse presenting with myeloneuropathy and mimicking Guillain-Barre syndrome. Intractable Rare Dis Res. 2020; 9(1):54-57.

2.Al-Sadawi M, Claris H, Archie C, et al. Inhaled nitrous oxide 'Whip-Its!' causing subacute combined degeneration of spinal cord. Am J Med Case Rep. 2018; 6(12): 237-240.

3.Alt RS, Morrissey RP, Gang MA, et al. Severe myeloneuropathy from acute high-dose nitrous oxide (N2O) abuse. J Emerg Med. 2011; 41(4):378-380.

4.Anderson D, Beecher G, van Dijk R, et al. Subacute combined degeneration from nitrous oxide abuse in a patient with pernicious anemia. Can J Neurol Sci. 2018; 45: 334-335.

5.Anderson M, Hollatz T. "Whippet" into your differential. Chest Conference;2015 Oct; Montreal, QC Canada.

6.Antezana A, Antezana Siles A, Hidalgo G. Toxic myelopathy secondary to recreational nitrous oxide abuse. 22nd World Congress of Neurology; 2015 Oct 15; Santiago, Chile.

7.Antonucci MU. Subacute combined degeneration from recreational nitrous oxide inhalation. J Emerg Med. 2018; 54(5): e105-e107.

8.Arshi B, Shaw S. Subacute ascending numbness. Clin Toxicol (Phila). 2014; 52(8): 905-906.

9.Brosset C, Nguyen Ngoc T, Nguyen Quang H,et al. No laughing matter! Revue

Neurologique. 2020; 176(5):401-402.

10.Buizert AR, Sharma R, Koppen H. When the Laughing Stops: Subacute Combined Spinal Cord Degeneration Caused by Laughing Gas Use. J Addict Med. 2017; 11(3): 235-236.

11.Butzkueven H, King JO. Nitrous oxide myelopathy in an abuser of whipped cream bulbs. J Clin Neurosci. 2000; 7(1):73-75.

12.Campdesuner V, Teklie Y, Alkayali T, et al. Nitrous oxide-induced vitamin B12 deficiency resulting in myelopathy. Cureus. 2020;12(7): e9088.

13.Cartner M, Sinnott M, Silburn P. Paralysis caused by "nagging". Med J Aust. 2007; 187(6):366-367.

14.Chen HJ, Huang CS. Nitrous oxide-induced subacute combined degeneration presenting with dystonia and pseudoathetosis: a case report. Acta Neurol Taiwan. 2016; 25: 50-55.

15.Cheng HM, Park JH, Hernstadt D. Subacute combined degeneration of the spinal cord following recreational nitrous oxide use. BMJ Case Rep. 2013:bcr2012008509.

16.Chiang TT, Hung CT, Wang WM, et al. Recreational nitrous oxide abuse-induced vitamin B12 deficiency in a patient presenting with hyperpigmentation of the skin. Case Rep Dermatol. 2013; 5(2):186-191.

17.Choi C, Kim T, Park KD, et al. Subacute combined degeneration caused by nitrous oxide intoxication: a report of two cases. Ann Rehabil Med. 2019; 43 (4): 530-534.

18.Chomin J, Nogar J. Subacute combined degeneration following chronic nitrous oxide use. 15th Annual Scientific Meeting of the American College of Medical

Toxicology; 2018; United States.

19.Demas A, Le Boisselier R, Simonnet L, et al. Another extensive nitrous oxide (N2O) myelopathy: The wood for the trees? Revue Neurologique.2020; 176 (4):293-295.

20.Diamond AL, Diamond R, Freedman SM, et al. "Whippets"-induced cobalamin deficiency manifesting as cervical myelopathy. J Neuroimaging. 2004; 14(3):277-280.

21.Dong X, Ba F, Wang R, et al. Imaging appearance of myelopathy secondary to nitrous oxide abuse: a case report and review of the literature. Int J Neurosci. 2019; 129(3): 225-229.

22.Duque MA, Kresak JL, Falchook A, et al. Nitrous oxide abuse and vitamin B12 action in a 20-year-old woman: a case report. Lab Med. 2015; 46(4):312-315.

23.Edigin E, Ajiboye O, Nathani A. Nitrous oxide-induced B12 deficiency presenting with myeloneuropathy. Cureus. 2019;11(8): e5331.

24.Egan W, Steinberg E, Rose J. Vitamin B12 deficiency-induced neuropathy secondary to prolonged recreational use of nitrous oxide. Am J Emerg Med. 2018; 36(9): 1717.e1-1717.e2.

25.Ernst LD, Brock K, Barraza LH, et al. Longitudinally extensive nitrous oxide myelopathy with novel radiographic features. JAMA Neurol. 2015; 72(11): 1370-1371.

26.Fang X, Li W, Gao H, et al. Skin hyperpigmentation: a rare presenting symptom of nitrous oxide abuse. Clin Toxicol. 2020;58(6): 476-481.

27.Fernandez D, Fara MG, Biary R, et al. Clinical reasoning: A 27-year-old man with unsteady gait. Neurology. 2017; 89(10): e120-123.

28.Fong VH, Vieira A. Neurological manifestations and electrophysiological studies in

acute toxicity of laughing gas. 68th American Academy of Neurology Annual Meeting; 2016; Vancouver, BC Canada.

29.Forzani BR, Lombardo SR, Ragucci M. Development of psychosis and subacute combined degeneration in a patient with pernicious anemia secondary to nitrous oxide abuse A case report. 2013.

30.Francis A, Crossley R, Brady S. Subacute progressive sensorimotor symptoms. Bmj 2019; 365: l1923.

31.Ghobrial GM, Dalyai R, Flanders AE, et al. Nitrous oxide myelopathy posing as spinal cord injury. J Neurosurg Spine. 2012; 16(5):489-491.

32.Glijn NHP, van der Linde D, Ertekin E, et al. Is nitrous oxide really that joyful? Neth J Med. 2017; 75(7): 304-306.

33.Hew A, Lai E, Radford E. Nitrous oxide abuse presenting with acute psychosis and peripheral neuropathy. Aust N Z J Psychiatry. 2018; 52(4): 388.

34.Hirvioja J, Joutsa J, Wahlsten P, et al. Recurrent paraparesis and death of a patient with 'whippet' abuse. Oxf Med Case Reports. 2016;2016(3): 41-43.

35.Hsu CK, Chen YQ, Lung VZ, et al. Myelopathy and polyneuropathy caused by nitrous oxide toxicity: a case report. Am J Emerg Med. 2012; 30(6):1016.e3-6.

36.Hu MH, Huang GS, Wu CT, et al. Nitrous oxide myelopathy in a pediatric patient. Pediatr Emerg Care. 2014; 30(4): 266-267.

37.Huang MY, Chang WH. Nitrous oxide-induced polyneuropathy in a teenager. Emerg Med J. 2009; 26(3):186.

38.Iwata K, O'Keefe GB, Karanas A. Neurologic problems associated with chronic

nitrous oxide abuse in a non-healthcare worker. Am J Med Sci. 2001; 322(3):173-174.
39.Ji R, Xie Z, Wang K, et al. Glucocorticoid treatment of myeloneuropathy induced by nitrous oxide toxicity. Neurol India. 2018; 6(4): 1167-1169.
40.Jiang J, Shang X. Clinical-radiological dissociation in a patient with nitrous oxide-induced subacute combined degeneration: A case report. BMC Neurology. 2020;20(1):99.
41.Johnson K, Mikhail P, Kim MG, et al. Recreational nitrous oxide-associated neurotoxicity. J Neurol Neurosurg Psychiatry. 2018; 89(8): 897-898.
42.Jones M, Ferris JA, Taylor RS. Progressive lower extremity weakness due to nitrous oxide induced myelopathy: A case report. 2015 Annual Assembly of the American Academy of Physical Medicine and Rehabilitation; 2015 Sep; Boston, MA United States.
43.Kanin M, Nguyen J, Rotblatt M. Say N2O to drugs: a unique presentation of symptomatic vitamin B12 deficiency. Am J Med.2020; 133(4): e145-e146.
44.Kaski D, Kumar P, Murphy E, et al. Iatrogenic B12-deficient peripheral neuropathy following nitrous oxide administration for functional tonic leg spasm: A case report. Clin Neurol Neurosurg. 2017; 160: 108-110.
45.Kwon YJ, Rho JH, Hwang J, et al. Unhappy end of 'happy balloons': subacute combined degeneration caused by nitrous oxide gas. J Clin Neurol. 2019;15(1): 118-119.
46.Lee HL, Lee SJ, Nam TS, et al. Acute cervical myelopathy following laughing gas abuse. Chonnam Med J. 2019; 55(2): 118-119.

47.Lin CY, Guo WY, Chen SP, et al. Neurotoxicity of nitrous oxide: multimodal evoked potentials in an abuser. Clin Toxicol (Phila). 2007; 45(1):67-71.

48.Lin RJ, Chen HF, Chang YC, et al. Subacute combined degeneration caused by nitrous oxide intoxication: case reports. Acta Neurol Taiwan. 2011; 20(2):129-137.

49.Lundin MS, Cherian J, Andrew MN, et al. One month of nitrous oxide abuse causing acute vitamin B 12 deficiency with severe neuropsychiatric symptoms. BMJ Case Rep. 2019; 12(2): e228001.

50. Mancke F, Kaklauskaite G, Kollmer J, et al. Psychiatric comorbidities in a young man with subacute myelopathy induced by abusive nitrous oxide consumption: a case report. Subst Abuse Rehabil. 2016; 7: 155-159.

51.Massey TH, Pickersgill TT, Peall KJ. Nitrous oxide misuse and vitamin B12 deficiency. BMJ Case Rep. 2016; 2016: bcr2016215728.

52.McArdle DJT, Gaillard F. Pernicious azotaemia? A case series of subacute combined degeneration of the cord secondary to nitrous oxide abuse. Journal of Clinical Neuroscience. 2020; 72:277-280.

53.Middleton JA, Roffers JA. Peripheral Neuropathy due to recreational use of nitrous oxide presenting after an ankle sprain with foot drop. Orthopedics. 2018; 41(1): e432-433.

54.Miller MA, Martinez V, McCarthy R, et al. Nitrous oxide "whippit" abuse presenting as clinical B12 deficiency and ataxia. Am J Emerg Med. 2004; 22(2):124.

55.Morris N, Lynch K, Greenberg SA. Severe motor neuropathy or neuronopathy due to nitrous oxide toxicity after correction of vitamin B12 deficiency. Muscle Nerve. 2015;

51(4): 614-616.

56.Morrissey RP, Alt RS, Howland MA, et al. Sub-acute high-dose nitrous oxide (N2O) exposure produces severe peripheral neuropathy. Abstracts of the XXIX International Congress of the European Association of Poison Centres and Clinical Toxicologists;2009; Stockholm, Sweden.

57.Ng J, Frith R. Nanging. Lancet. 2002; 360(9330):384.

58.Olney A, Kanakeswaran G, Venkatesh M, et al. Axonal neuropathy, myelopathy and hypercoagulable state secondary to chronic nitrous oxide abuse despite combined b12 supplementation. 71st Annual Meeting of the American Academy of Neurology;2019; United States.

59.Onrust MR, Frequin ST. Subacute combined spinal cord degeneration by recreational laughing gas (N_2O) use. J Cent Nerv Syst Dis. 2019; 11:1179573519838277.

60.Papathanasiou A, Ham T, Cope T, et al. Nitrous oxide recreational use and subacute combined degeneration. 1st Congress of the European Academy of Neurology; 2015; Berlin, Germany.

61.Pema PJ, Horak HA, Wyatt RH. Myelopathy caused by nitrous oxide toxicity. AJNR Am J Neuroradiol. 1998 ; 19(5):894-896.

62.Prigge D, King A, Menke N, et al. Normal MRI with symptoms and diagnosis of subacute combined degeneration from nitrous oxide abuse. 2012 Annual Meeting of the North American Congress of Clinical Toxicology (NACCT) October 1–6, 2012 Las Vegas, NV, USA.

63.Pugliese RS, Slagle E J, Oettinger G R, et al. Subacute combined degeneration of the spinal cord in a patient abusing nitrous oxide and self-medicating with cyanocobalamin. Am J Health Syst Pharm. 2015; 72(1): 952-957.

64.Rheinboldt M, Harper D, Parrish D, et al. Nitrous oxide induced myeloneuropathy: a case report. Emerg Radiol. 2014; 21(1):85-88.

65.Richardson PG. Peripheral neuropathy following nitrous oxide abuse. Emerg Med Australas. 2010; 22(1):88-90.

66.Sadr PM. "Whippets" The clinical manifestations of nitrous oxide induced subacute combined degeneration.Poster Abstracts from the AAAP 20th Annual Meeting and Symposium. 2010.

67.Seed A, Jogia M. Lessons of the month: Nitrous oxide-induced functional vitamin B12 deficiency causing subacute combined degeneration of the spinal cord. Clinical Medicine, Journal of the Royal College of Physicians of London. 2020; 20 (3): e7-9.

68.Shah K, Murphy C. Nitrous oxide toxicity: case files of the Carolinas Medical Center Medical Toxicology Fellowship. Journal of Medical Toxicology. 2019;15 (4): 299-303.

69.Shen Q, Lu H, Wang H, et al. Acute cognitive disorder as the initial manifestation of nitrous oxide abusing: a case report. Neurological Sciences. 2019.

70.Shulman RM, Geraghty TJ, Tadros M. A case of unusual substance abuse causing myeloneuropathy. Spinal Cord. 2007; 45(4):314-317.

71.Simpson A, Rinker J, Rushton WF. Intravenous immunoglobulin (IVIG) use in subacute combined degeneration secondary to vitamin B12 deficiency from nitrous

oxide abuse. 39th International Congress of the European Association of Poisons Centres and Clinical Toxicologists; 2019; Italy.

72.Sleeman I, Wiblin L, Burn D. An unusual cause of falls in a young woman. J R Coll Physicians Edinb.2016; 46: 160-162.

73.Sotirchos ES, Saidha S, Becker D. Neurological picture. Nitrous oxide-induced myelopathy with inverted V-sign on spinal MRI. J Neurol Neurosurg Psychiatry. 2012; 83(9):915-916.

74.Spickler M. Distal symmetric peripheral polyneuropathy due to nitrous oxide use. American Academy of Physical Medicine and Rehabilitation Annual Meeting; 2019; United States.

75.Stockton L, Simonsen C, Seago S. Nitrous oxide–induced vitamin B12 deficiency.Proc (Bayl Univ Med Cent).2017; 30(2): 171-172.

76.Sutaria J, Volosin A, Garza J, et al. Whippit abuse: Not a laughing matter.Hospital Medicine; 2018; United States.

77.Tatum WO, Bui DD, Grant EG, et al. Pseudo-guillain-barre syndrome due to "whippet"-induced myeloneuropathy. J Neuroimaging. 2010; 20(4):400-401.

78.Thompson AG, Leite MI, Lunn MP, et al. Whippits, nitrous oxide and the dangers of legal highs. Pract Neurol. 2015; 15(3): 207-209.

79.Waclawik AJ, Luzzio CC, Juhasz-Pocsine K, et al. Myeloneuropathy from nitrous oxide abuse: unusually high methylmalonic acid and homocysteine levels. WMJ. 2003; 102(4):43-45.

80.Waters MF, Kang GA, Mazziotta JC, et al.Nitrous oxide inhalation as a cause of

cervical myelopathy. Acta Neurol Scand. 2005; 112(4):270-272.

81.Weng HY, Lin CY. N2O Intoxication-caused subacute combined degeneration of spinal cord-an unusual case with paraplegia and autonomic dysfunction. 23rd World Congress of Neurology; 2017; Japan.

82.Williamson J, Huda S, Damodaran D. Nitrous oxide myelopathy with functional vitamin B 12 deficiency. BMJ Case Rep 2019;12(2): e227439.

83.Wu MS, Hsu YD, Lin JC, et al. Spinal myoclonus in subacute combined degeneration caused by nitrous oxide intoxication. Acta Neurol Taiwan. 2007; 16(2):102-105.

84.Yuan JL, Wang SK, Jiang T, et al. Nitrous oxide induced subacute combined degeneration with longitudinally extensive myelopathy with inverted V-sign on spinal MRI: a case report and literature review. BMC Neurol. 2017;17(1): 222.

85. Zhao B, Zhao L, Li Z, et al. Subacute combined degeneration induced by nitrous oxide inhalation: Two case reports. Medicine. 2020; 99(18): e19926.
